# Multifocal Orthokeratology versus Conventional Orthokeratology for Myopia Control: A Paired-Eye Study

**DOI:** 10.3390/jcm10030447

**Published:** 2021-01-24

**Authors:** Martin Loertscher, Simon Backhouse, John R. Phillips

**Affiliations:** 1 School of Optometry and Vision Science, The University of Auckland, Auckland 1023, New Zealand; martin.loertscher@fhnw.ch; 2 Institute für Optometrie, Fachhochschule Nordwestschweiz, 4600 Olten, Switzerland; 3 School of Medicine—Optometry, Deakin University, Geelong, VIC 3220, Australia; simon.backhouse@deakin.edu.au; 4 Department of Optometry, Asia University, Taichung 41354, Taiwan

**Keywords:** myopia control, myopia progression, orthokeratology, multifocal optics, eye length

## Abstract

We conducted a prospective, paired-eye, investigator masked study in 30 children with myopia (−1.25 D to −4.00 D; age 10 to 14 years) to test the efficacy of a novel multifocal orthokeratology (MOK) lens compared to conventional orthokeratology (OK) in slowing axial eye growth. The MOK lens molded a center-distance, multifocal surface onto the anterior cornea, with a concentric treatment zone power of +2.50 D. Children wore an MOK lens in one eye and a conventional OK lens in the fellow eye nightly for 18 months. Eye growth was monitored with non-contact ocular biometry. Over 18 months, MOK-treated eyes showed significantly less axial expansion than OK-treated eyes (axial length change: MOK 0.173 mm less than OK; *p* < 0.01), and inner axial length (posterior cornea to anterior sclera change: MOK 0.156 mm less than OK, *p* < 0.01). The reduced elongation was constant across different baseline progression rates (range −0.50 D/year to −2.00 D/year). Visual acuity was less in MOK vs. OK-treated eyes (e.g., at six months, MOK: 0.09 ± 0.01 vs. OK: 0.02 ± 0.01 logMAR; *p* = 0.01). We conclude that MOK lenses significantly reduce eye growth compared to conventional OK lenses over 18 months.

## 1. Introduction

The prevalence of myopia is increasing worldwide [1,2] and has reached epidemic levels in parts of Asia [3,4]. The abnormal enlargement of the eye, which is the structural basis of the myopic refractive error, typically begins in childhood and progresses until early adulthood. The progressive eye enlargement also causes the tissues of the eye to become stretched and damaged [5], making myopia a significant risk factor for sight-threatening conditions such as glaucoma, retinal detachment, and myopic maculopathy later in life [6]. Since the relative risk of developing these conditions increases sharply as myopia progresses to higher degrees [7], there is significant interest in controlling myopia progression during childhood, when the rate of eye enlargement is typically highest [8].

A variety of methods for controlling myopia progression is available [9], including nightly instillation of atropine eye drops, multifocal contact lenses, defocus incorporated (DIMS) spectacles, and orthokeratology. The relative efficacy of these methods in slowing eye enlargement in the longer term is controversial and difficult to assess [10]. However, none of these methods come close to totally eliminating myopia progression in the longer term, and so there is significant room for improving efficacy of these methods.

Several studies have investigated the effect of combining low-dose atropine eye drops with orthokeratology in attempts to enhance efficacy. Recent meta-analyses of these studies [11,12,13] have concluded that this combination can significantly improve efficacy in slowing progression relative to the efficacy of orthokeratology alone.

The aim of this study was to investigate whether combining two optical approaches to myopia control, orthokeratology and multifocal optics, might improve efficacy. We have compared the efficacy of a novel multifocal orthokeratology (MOK) lens with that of conventional orthokeratology (OK) in controlling myopia progression in children. The MOK lens molds concentric multifocal optical elements onto the surface of the cornea. We have tested its efficacy in a paired-eye study in which the MOK lens is worn in one eye and a conventional OK lens is worn in the other nightly for 18 months. The results of our study show that the MOK lens is significantly more effective than conventional orthokeratology in slowing childhood myopia progression over this period.

## 2. Experimental Section

The study adhered to the tenets of the Declaration of Helsinki, received approval from the New Zealand Health and Disability Ethics Committee (Approval No. LRS11/02/001), and was prospectively registered with the Australian and New Zealand Clinical Trial Registry (ANZCTR Number: ACTRN12611000499987). Participants gave written consent (parents) and written assent (children) to participate. Lenses and solutions were provided at no cost to all participants.

### 2.1. Study Participants and Recruitment

A total of 30 children were recruited into the study. Inclusion criteria were as follows:

- Age: between 10 and 14 years at the time of enrolment.
- Refractive error: subjective refraction between −1.25 and −4.00 D spherical equivalent (sphere + ½ cylinder) in both eyes.
- Myopia progression: demonstrated progression of at least −0.50 D in the year prior to enrolment, based on past records.
- Visual acuity (VA): best-corrected, high-contrast, Snellen VA of 6/6 (0.0 logMAR) or better in both eyes.

Exclusion criteria were as follows:

- Astigmatism greater than −1.50 D, because only spherical corrections were applied with OK and MOK lenses.
- Prior or current myopia control treatment (pharmaceutical or optical, including orthokeratology) or RGP lens wear.
- Anterior eye pathology (assessed by slit-lamp biomicroscopy at the initial eye exam), or previous corneal surgery, or past or current ocular medications, or relevant, pre-existing systemic conditions (e.g., diabetes).
- Amblyopia or strabismus.
- Anisometropia ≥ 1.00 D, because significant pre-existing anisometropia may have confounded the comparisons made between eyes with this paired-eye control study design.

### 2.2. Study Design

To compare the efficacy of MOK vs. OK treatments in slowing eye elongation, we used a randomized, investigator-masked, longitudinal, paired-eye study design over 18 months. The thirty participants were pseudo-randomly assigned to two groups using a permuted-block design, with random block sizes of four or six. Randomization was stratified by gender and ethnicity (East-Asian and Non-East Asian which included New Zealand European, Indian, and Maori/Pasifika). Participants in one group wore the MOK lens overnight in the dominant eye, and those in the other group wore the MOK lens in the non-dominant eye. All participants wore a conventional OK lens in the fellow eye. Eye dominance was determined using a simple sighting test [14].

#### Outcome Measures and Data Analysis

Masked examiners, who were not involved in the contact lens fitting or management of the participants, made the primary outcome measures on both eyes of each participant. The main outcome measure was elongation of the eye over a period of 18 months. A non-contact optical low-coherence reflectometer (Lenstar LS 900, Haag-Streit AG, Köniz, Switzerland) was used to obtain the average of five consecutive ocular biometry measures in each eye and at each measurement visit. This allowed computation of axial length (AL; anterior cornea to retinal pigment epithelium (RPE)), anterior chamber depth (ACD; anterior cornea to anterior crystalline lens), vitreous chamber depth (VCD; posterior crystalline lens to RPE), inner axial length (IAL; posterior cornea to anterior sclera) and choroidal thickness (ChT; RPE to anterior sclera).

Secondary outcome measures included visual acuity (VA) measured with a high-contrast logMAR acuity chart (Medmont Pty, Nunawading, VIC, Australia), contrast sensitivity (CS) measured using a Pelli–Robson chart [15] and stereoacuity measured at 40 cm distance with the FLY Stereo Acuity Test, using the polarized Verhoeff circles (Vision Assessment Corporation, Elk Grove Village, IL, USA). Pupil diameters were measured at baseline with an infrared pupilometer (VIP 200586009, NeurOptics Inc, Laguna Hills, CA, USA) under photopic (800 lux) conditions during distance viewing. Non-cycloplegic refractions were made with an infrared open field autorefractor (NVision-K 5001, Shin-Nippon, Tokyo, Japan) both on-axis and peripherally across the horizontal visual field from 35° nasal to 35° temporal in both eyes. Refractions were made with a +1.50 D lens placed over the contralateral eye to control accommodation. Peripheral refractions were converted to power vectors [16] and plotted as actual (not relative) values. However, autorefractor measures made through multifocal optics as in this study should be interpreted with caution [17]. All primary and secondary outcome measures were performed at baseline (BL) before fitting either eye with a lens, then after 3–4 weeks of successful lens wear; these are referred to here as Outcome Measure 0 (OM0). Further outcome measures were made at 6 months, (OM6), 12 months (OM12) and 18 months (OM18) after OM0.

Data analysis was conducted using SPSS Statistics Version 19 (IBM, Armonk, NY, USA). The data were first checked for normality, and data following a normal distribution were analyzed using a repeated-measures general linear model (RGLM) with Bonferroni post-hoc correction for multiple pairwise comparisons. Results were reported as statistically significant when *p* ≤ 0.05. When the data violated the sphericity assumption, the Greenhouse–Geisser-corrected results were reported. Non-normally distributed data were analyzed with the equivalent non-parametric test (Friedman test and Wilcoxon signed-rank test).

### 2.3. Lens Design, Manufacture, and Fitting

**Figure 1:**
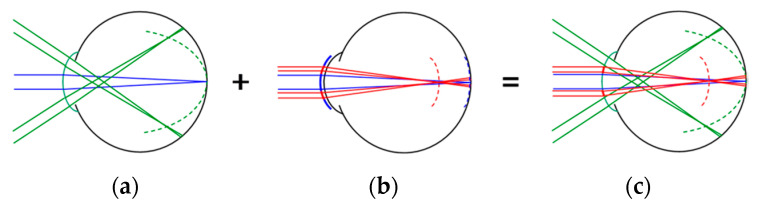
Summary optical concept of the multifocal orthokeratology (MOK) lens. (**a**) Conventional orthokeratology induces peripheral myopic defocus while correcting on-axis refractive error. (**b**) Dual-focus optics creates simultaneous, on-axis myopic retinal defocus. (**c**) The multifocal treatment zone is molded onto the corneal surface. The MOK lens combines both optical concepts.

**Figure A1:**
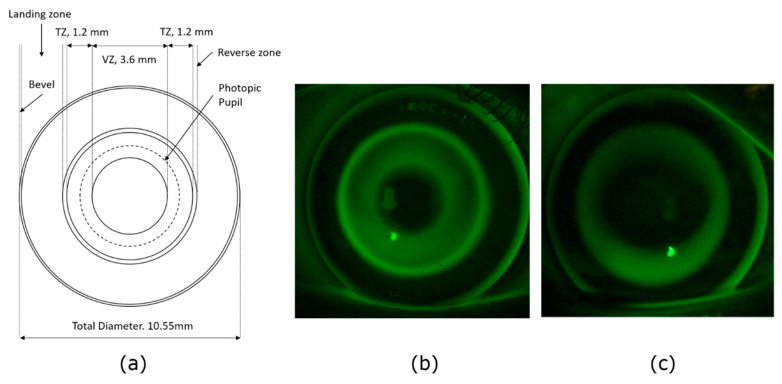
Details of the multifocal orthokeratology (MOK) Lens. (**a**) Diagram of the multifocal orthokeratology (MOK) lens. Vision correction zone (VZ): Diameter (MOK: 3.6 mm; OK: 6.00 mm), back surface radius based on topography and amount of myopia to be corrected. Treatment zone (TZ) annulus width 1.2 mm. TZ power: +2.50 D relative to VZ power. Reverse zone: Individually made based on topography to connect TZ with the landing zone. Landing zone: Based on topography. The landing zone was needed to achieve good lens centration, with an average back surface radius of 7.79 mm. Total lens diameter was 1 mm less than the horizontal corneal diameter. Bevel (edge lift). The conventional orthokeratology lenses used as controls were identical but without the treatment zone (i.e., the VZ had a diameter of 6 mm and extended out to the reverse zone). (**b**) Example MOK lens Fluorescein pattern (**c**) Example OK lens (control) Fluorescein pattern.

The novel MOK lens was designed to modify the power profile of the cornea (Figure 1 and Appendix A Figure A1a) such that paraxial rays entering the pupil formed two focal planes: one located conjugate with the retina to provide clear vision, and the other located anterior to the retina, creating +2.50 D of myopic retinal defocus.

As shown in Figure A1a, the central vision correction zone (VZ) of the MOK lens corrected the refractive error and had a diameter of 3.60 mm. The surrounding treatment zone (TZ) had an annulus width of 1.20 mm. The refractive power of the TZ was 2.50 D more positive than the power of the VZ. Examples of fluorescein patterns for MOK and OK lenses are shown in Figure A1b,c. Lenses for each participant were manufactured by Falco Linsen AG, Tägerwilen, Switzerland, based on the participant’s corneal topography maps (E300, Medmont Pty Ltd., Nunawading, VIC, Australia), subjective refraction and horizontal corneal diameter. Both MOK and conventional OK lenses (controls) were lathe-cut in Boston XO (Hexafocon A) with a Dk value of 100. The lenses were tinted Red (for Right eye) and Lilac (for Left eye) to prevent inadvertent cross-over.

## 3. Results

### 3.1. Participant Demographics and Baseline Measures

Overall, of the 30 children recruited, 15 identified as East Asian, and 20 were female. Mean age was 12.2 ± 1.3 years. MOK lenses were allocated to 14 dominant eyes and 16 non-dominant eyes. Mean stereoacuity was 25 ± 8 arcsec before fitting lenses. As expected with this paired-eye study design, baseline visual acuity, contrast sensitivity, photopic pupil diameter, refraction, and ocular biometry measures were not significantly different between eyes assigned to wear MOK lenses and those assigned to OK lenses (Table 1). The primary outcome measure was myopia progression (measured as eye elongation) over 18 months. Importantly, the paired-eye study design naturally tends to match experimental and control eyes for myopia progression at baseline, as each experimental eye is matched with a control eye from the same individual. Table 1 confirms that baseline progression for MOK- and OK-treated eyes was not significantly different.

Of the 30 children enrolled in the study, 28 completed the 18-month outcome measures and two dropped out. One left the study before the 6-month visit. The other moved overseas after the 6-month measures.

### 3.2. Lens Fitting

Typically, achieving a satisfactory fit of the lens on the eye took more than one attempt. Conventional OK lenses required an average of 2.2 ± 1.1 lenses to achieve a successful fit, whereas MOK lenses required an average of 2.5 ± 1.3 lenses. It is important to emphasize that when a lens needed changing (e.g., because the fit was unsatisfactory), lens wear was stopped in both eyes. On average, it took about 26 days (range 6 to 48 days) between trialing the first lens and obtaining a successful fit. This was partly due to delivery times early in the study, as the lenses were manufactured in Switzerland but fitted in New Zealand. This was rectified later in the study with express delivery.

### 3.3. Changes in Axial Biometry Measures Over Time

#### 3.3.1. Short-Term Changes

**Figure 2:**
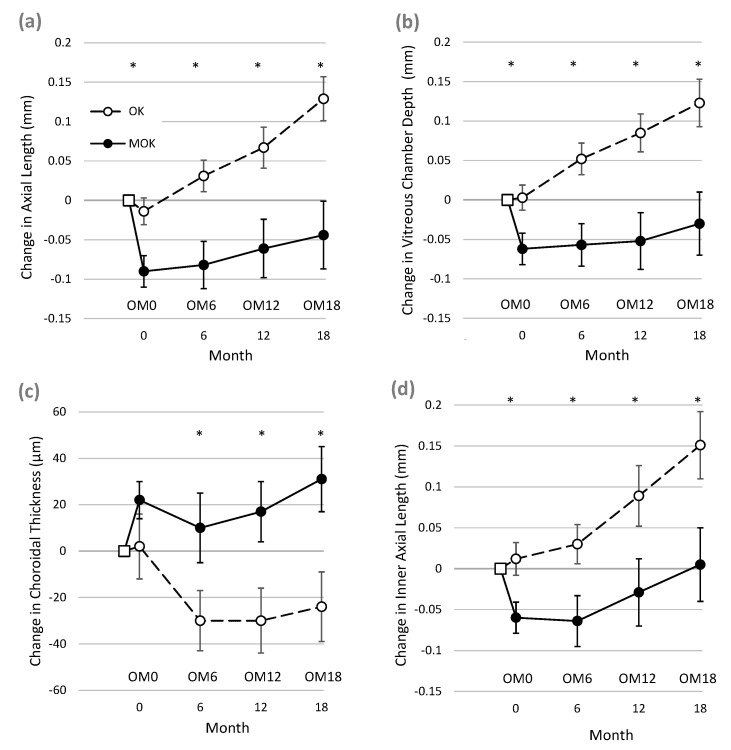
Changes in ocular dimensions during the study in eyes fitted with MOK lenses (solid lines and symbols) and eyes fitted with conventional orthokeratology (OK) lenses (dashed lines and open symbols). In each panel, the open square symbol represents baseline, and OM0, OM6, OM12, and OM18 indicate when outcome measures were taken. Changes over time in (**a**) axial eye length (AL), (**b**) vitreous chamber depth (VCD), (**c**) choroidal thickness (ChT), (**d**) inner axial length (IAL: posterior cornea to anterior sclera). * indicates significant difference in values for MOK and OK-treated eyes, (*p* < 0.05; repeated-measures general linear model with Bonferroni post-hoc correction (**a**,**b**,**d**) or Wilcoxon signed-rank test (**c**)). Error bars show ± 1 S.E.M.

After only 3–4 weeks of successful lens wear, eyes wearing MOK lenses showed markedly different biometric changes compared to eyes wearing conventional OK lenses (see Figure 2, period between BL and OM0). Pairwise comparison showed that in MOK-treated eyes, AL, VCD, and IAL had all shortened significantly when compared to OK-treated eyes.

Mean short-term treatment differences (BL to OM0) were as follows: AL = −0.076 mm (95% CI −0.197 to −0.043 mm, *p* = 0.00016); VCD = −0.065 mm (95% CI −0.0820 to −0.035 mm, *p* = 0.00019); and IAL = −0.081 mm (95% CI −0.099 to −0.032 mm, *p* = 0.0003). The data for ChT did not follow a normal distribution, but pairwise comparisons of ChT between MOK and OK eyes using the Wilcoxon signed-rank test showed no significant difference between ChT change in OK and MOK eyes at time OM0 (difference = 0.019 ± 0.07 mm, *p* = 0.072).

#### 3.3.2. Longer-Term Changes

##### Changes between Baseline and 18 Months

Figure 2 and Table 2 show that after 18 months of lens wear, increases in AL, VCD, and IAL were significantly less in eyes wearing MOK lenses than in eyes wearing OK lenses. In addition, ChT increased significantly more in MOK-treated eyes than in OK-treated eyes. In addition, CCT thinned significantly less in MOK- than OK-treated eyes (Table 2). In this analysis, neither pupil diameter nor ocular dominance showed significant interactions with these changes (all *p* > 0.11).

**Figure 3:**
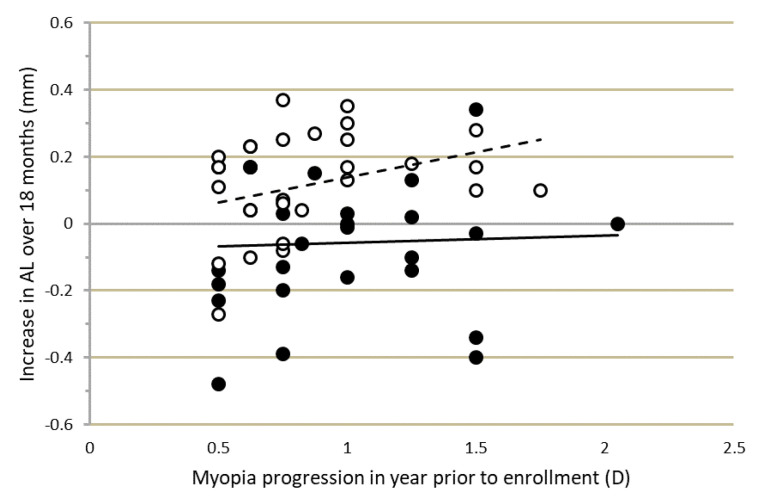
Correlation between the change in axial length (AL) over 18 months of lens wear vs. myopia progression in the year prior to enrollment. Filled circles and solid line: multifocal orthokeratology (MOK) treated eyes: *n* = 28, R^2^ = 0.002, *p* = 0.83. Open circles and dashed line: orthokeratology (OK) treated eyes: *n* = 28, R^2^ = 0.114, *p* = 0.08.

**Figure A2:**
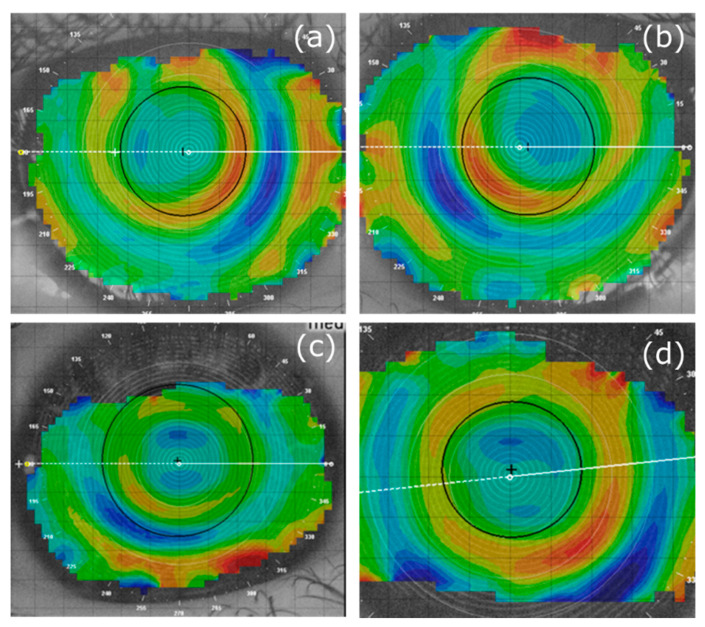
Tangential difference maps (18-month topography minus baseline topography). (**a**) Map for the MOK-treated eye with the greatest axial length increase over 18 months (+0.34 mm) and (**b**) map for the fellow OK-treated eye with an AL increase of +0.17 mm. (**c**) MOK-treated eye with the least axial length increase over 18 months (−0.48 mm) and (**d**) map for the fellow OK-treated eye with an AL increase of −0.27 mm.

The progression-inhibiting effect, particularly of MOK lens wear, was not related to the progression rate in the year prior to lens fitting. Figure 3 shows that the changes in AL over the course of the study were not correlated with initial progression rate, which varied markedly among participants. Similar effects were found for VCD and IAL. Figure 3 also shows that the change in AL over 18 months varied widely among participants. For MOK-treated eyes, change in AL over 18 months varied from +0.34 mm in the fastest progressing eye to −0.48 mm in the slowest progressing eye. Corneal topography (difference) maps for these MOK-treated eyes are shown in Figure A2a,c with topography maps for the fellow, OK-treated eyes of these participants shown in Figure A2b,d.

Figure 2 illustrates that for each biometric measure, much of the difference between the data for MOK- and OK-treated eyes at 18 months appears to be accounted for by the short-term changes occurring within the first few weeks of lens wear (i.e., between BL and OM0) as described above. Therefore, it was of interest to determine whether elongation rates over the rest of the study (i.e., after the short-term effects) were different between the two eyes. To achieve this, the changes in AL, VCD, and IAL occurring between OM0 and OM18 were compared between MOK-treated eyes and OK-treated eyes.

##### Changes Between OM0 and OM18

In this analysis, RGLM was used to re-analyze the data with OM0 used as the new baseline. We found a significant overall effect of lens type on VCD (F _1.0, 50.0_ = 5.383, *p* = 0.024). Bonferroni-corrected post-hoc pairwise comparisons showed significantly less VCD elongation in MOK-treated eyes compared to OK-treated eyes at 12 months (−0.067 ± 0.12 mm, *p* = 0.028) and 18 months (−0.088 ± 0.14 mm, *p* = 0.024), although at 6 months, there was no significant difference (−0.038 ± 0.11 mm, *p* = 0.077). There was also a significant overall effect of lens type on AL (F _1.0, 50.0_ = 4.568, *p* = 0.037). Bonferroni-corrected post-hoc pairwise comparisons showed significantly less AL elongation in MOK-treated eyes compared to OK-treated eyes at 18 months (−0.097 ± 0.15 mm, *p* = 0.013), although the differences at 6 months (−0.033 ± 0.11 mm, *p* = 0.116) and 12 months (−0.050 ± 0.13 mm, *p* = 0.132) were not significant. There was no significant overall effect of lens type on IAL (mean difference −0.031 mm (95% CI −0.077 to 0.016) F _1.0, 46.0_ = 1.731, *p* = 0.195).

### 3.4. Peripheral Refractions

**Figure A3:**
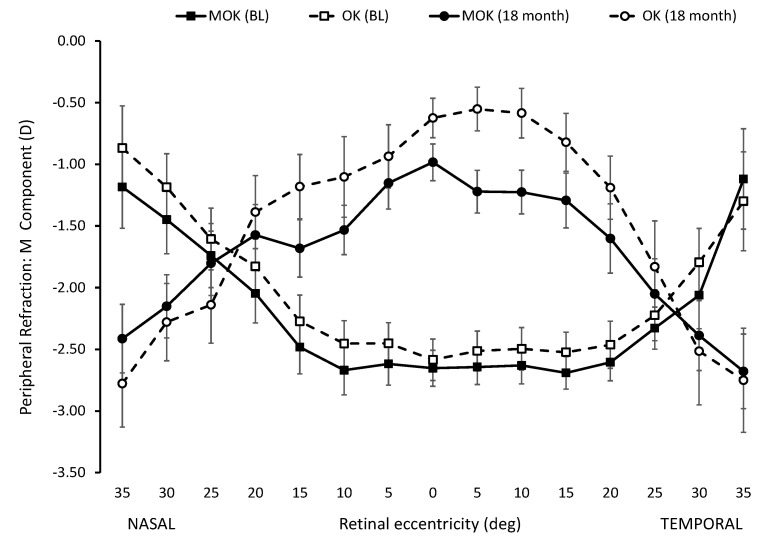
Peripheral refractions at eccentricities between 35° nasal and 35° temporal retina. Square symbols show measures made at baseline, before any lens wear (filled squares: eyes assigned to wear MOK lenses, open squares: eyes assigned to wear OK lenses). Circles show measures made after 18 months of lens wear (filled circles: MOK-treated eyes, open circles: OK-treated eyes). Error bars: ±1 S.E.M.

**Figure A4:**
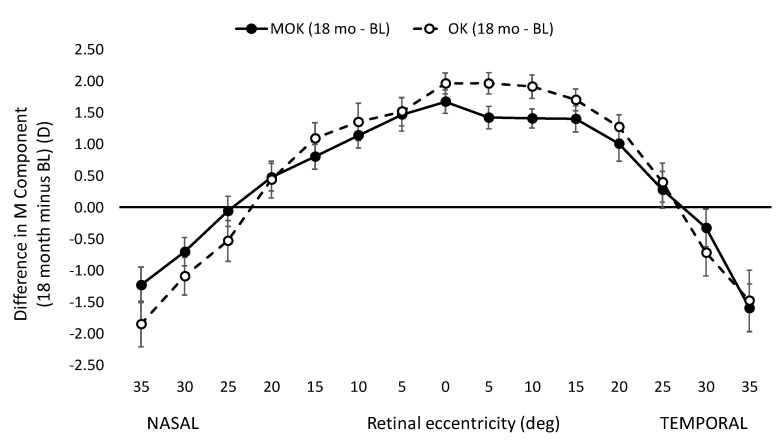
Changes in peripheral refractions between baseline (before lens wear) and after 18 months of lens wear for MOK-treated eyes (solid symbols and line) and OK-treated eyes (open symbols and dashed line). Error bars: ±1 S.E.M.

As expected, the peripheral refraction (M-component) profiles resulting from OK and MOK lens wear changed from relative hyperopia in the periphery (compared to on-axis refraction) before lens wear to relative myopia in the periphery (compared to on-axis refraction) after OK and MOK lens wear. These effects are apparent in the peripheral refraction profiles for baseline and after 18 months lens wear, as shown in Figure A3. Although the refraction profiles before lens wear were not different for eyes assigned to wear MOK and OK lenses, the profiles after lens wear were different: the profile for MOK-treated eyes tended to be more myopic than for OK-treated eyes. However, for these differences to qualify as a candidate explanation for the difference in the efficacy of the two lens types in slowing eye growth over 18 months, it is necessary to compare the refractive changes induced within each eye over the 18 months of the study. Figure A4 shows the induced peripheral refractive changes (18 months minus baseline values) for the MOK- and OK-treated eyes. There is no significant difference in these values at any retinal eccentricity apart from the values at 5° nasal, where the value for the MOK-treated eyes is significantly more myopic than that for the OK-treated eyes (*p* = 0.025, Mann–Whitney).

We also computed changes in J0 and J45 induced by each treatment over the 18 months of the study (i.e., 18 months minus baseline values). The values for J0 were not statistically different for MOK and OK treatment at any eccentricities apart from two. At 5° and 25° temporal retina, MOK treatment values were more negative by −0.26 D (95% CI −0.54 to 0.006, *p* = 0.037) and −0.94 D (95% CI −1.29 to 0.59, *p* = 0.00008), respectively. The values for J45 were not statistically different for MOK and OK treatments at any eccentricities apart from nasal 10°, where the MOK-treatment value was more positive by +0.286 D (95% CI −0.025 to 0.54, *p* = 0.006).

### 3.5. Visual Performance

There was a significant reduction in high-contrast VA in eyes corrected with MOK lenses (Friedmann test, *p* = 0.001). Multiple pairwise comparisons (Wilcoxon signed-rank test) showed that VA was significantly reduced from 0.01 ± 0.02 logMAR at BL (measured with spectacles) to 0.05 ± 0.01 logMAR at OM0 with MOK (*p* = 0.002). This corresponds to an acuity loss of 2.3 letters. However, no significant changes in VA in MOK-treated eyes were found between subsequent visits (*p* > 0.05).

In eyes fitted with OK lenses, no significant changes in VA were recorded at any point (*p* > 0.05). However, mean VA at BL (0.00 ± 0.02 logMAR, measured with spectacles) was 0.03 ± 0.01 logMAR at OM0 with corneal molding, corresponding to an acuity loss of 1.6 letters.

Between-eye comparison (MOK vs. OK) showed a better VA in OK-treated eyes at six months (at OM6: MOK: 0.09 ± 0.01 logMAR vs. OK: 0.02 ± 0.01 logMAR; *p* = 0.01) and at 12 months (at OM12: MOK: 0.09 ± 0.02 logMAR vs. OK: 0.03 ± 0.01 logMAR, *p* = 0.01). These differences in VA were not explained by simple changes in refraction over time, as there was no significant difference (Wilcoxon signed-rank test) in subjective over-refractions (spherical equivalent) between MOK and OK-treated eyes at any measurement visit (OM0, *p* = 0.491; OM6, *p* = 0.212; OM12, *p* = 0.337; OM18, *p* = 0.280).

For both MOK- and OK-treated eyes, contrast sensitivity (measured with a Pelli–Robson chart) decreased from baseline (MOK: 1.63, OK: 1.64) to (MOK: 1.57, OK: 1.58; *p* < 0.05) 6 months (OM6) and then increased again for both treatments. Pairwise comparison (Wilcoxon signed-rank test) between MOK- and OK-treated eyes showed no statistically significant difference at any time point from BL to OM18 (*p* > 0.01).

Non-parametric repeated-measures analysis of stereo vision (FLY Stereo Acuity Test) revealed no change in stereovision from BL (*p* = 0.329) in spite of the different treatment applied to each eye.

## 4. Discussion

We employed a paired-eye study design to compare myopia progression (measured as eye elongation) in eyes treated with a novel multifocal orthokeratology (MOK) lens vs. contralateral eyes treated with a conventional orthokeratology (OK) lens in 30 children over 18 months. Between baseline (before lens wear) and measures at 18 months, MOK-treated eyes elongated significantly less than OK-treated eyes (e.g., for AL, MOK-treated eyes elongated 0.173 mm less than OK-treated eyes). Moreover, in MOK-treated eyes, AL remained at baseline levels over the study period of 18 months. This degree of eye growth inhibition has generally only been reported with the use of 1% atropine eye drops [18], which have significant side effects associated with cycloplegia, mydriasis, and rebound on cessation of use. The most likely explanation for the increased efficacy of MOK treatment over conventional orthokeratology is the addition of on-axis, simultaneous myopic defocus caused by the multifocal optics within the pupil margin in MOK-treated eyes. Soft multifocal contact lenses have been demonstrated to slow myopia progression and eye growth in children compared to single vision contact lenses [19], and we believe that this effect has added to the known myopia-inhibiting effect of conventional OK [20].

Previous studies have typically used AL as the metric for change in eye size [21]. However, orthokeratology can affect AL (anterior cornea to RPE) by increasing choroidal thickness (ChT) [22] and decreasing central corneal thickness (CCT) [23]. Therefore, we have also used the inner axial length (IAL) measure (posterior cornea to anterior sclera) to describe changes in eye size unaffected by changes in the choroid or cornea. Our results for IAL essentially parallel those for AL and show that for IAL, MOK-treated eyes elongated 0.156 mm less than OK-treated eyes over 18 months.

Comparisons of changes in MOK- and OK-treated eyes are complicated by the finding that MOK-treated eyes rapidly shortened (between BL and OM0, in Figure 2), while OK-treated eyes did not. The explanation for this rapid shortening of AL in MOK-treated eyes is unclear, as it could not be fully accounted for by the increase in choroidal thickness (ChT) that was also observed in MOK-treated eyes over the same short time period. Mean ChT increased by 20 µm in MOK- vs. OK-treated eyes, but mean VCD decreased by 60 µm in MOK- vs. OK-treated eyes between BL and OM0. Moreover, a rapid reduction in IAL was also observed, indicating that factors other than changes in CCT or ChT were causing the reduction in eye size. It is notable that in some MOK-treated eyes, AL was reduced by large amounts between baseline and 18 months: Figure 3 shows that for four participants, AL was reduced between 0.34 and 0.48 mm, although for most MOK-treated eyes, this was less than 0.2 mm. Significant mean reductions in AL (≈0.2 mm) have also been reported after 1 year of nightly instillation of 1% atropine [18].

Once the sort-term changes had occurred, further gradual changes in eye length over the following 18 months also showed that elongation in MOK-treated eyes was significantly less than in OK-treated eyes (AL: −0.097 ± 0.15 mm, *p* = 0.013; VCD: −0.088 ± 0.14 mm, *p* = 0.024).

In this study, the myopia progression rate for each child in the year prior to enrollment was determined; it varied between −0.50 D and −2.00 D/year among participants. Comparison of change in eye length (AL) over the course of the study for different prior progression rates showed that there was no significant relationship between change in eye length and prior progression rate, particularly in MOK-treated eyes. The change in eye length over 18 months was essentially the same in children progressing at 0.50 D/year as in those progressing at 2.00 D/year at baseline (Figure 3). This implies that MOK lens wear (and to a lesser extent, OK lens wear) has a constant myopia-controlling effect rather than a proportional effect. This corroborates the findings of others [10] that in general, myopia control methods cause an absolute reduction rather than a percent reduction in progression. Eye growth is controlled by a retina–choroid–sclera signaling pathway [24], and it seems likely that this absolute, rather than proportional, effect of myopia control methods on progression may originate in this pathway. A recent study of the effects of atropine and optical defocus on the thickness of the choroid in children [25] demonstrated that the increase in choroidal thickness caused by 2.00 D of myopic retinal defocus was constant (+12 µm) among children, even though the baseline choroidal thickness varied markedly among children (range: 160 to 450 µm). A similar constant increase (+21 µm) in choroidal thickness was observed after 1 week of 0.3% atropine treatment in the same group of children.

Our data suggest that the reduced rate of eye enlargement in MOK-treated eyes compared to OK-treated eyes is unlikely to be accounted for by differences in the refractive state of the peripheral retina. We observed no significant difference in the change in peripheral refraction profiles (18-month minus baseline) for the two lens types between 35° and 5° nasal retina or between 35° and 20° temporal retina (Figure A4). However, we only measured peripheral refractions in the horizontal meridian, so accumulated small differences in peripheral refractions across the entire retina between MOK- and OK-treated eyes remain a possible explanation for the different efficacies of the two lens types. Measurements in several different meridians, as has been performed by others (e.g., [26]), could help resolve this limitation. However, autorefractor measures made through multifocal optics, as we have done in the current study, should be interpreted with caution. The aim of the multifocal optics was to simultaneously create two focal planes within the eye (see Figure 1), but the single refractive value produced by the autorefractor at each location is likely to be an average refractive value, which is measured across the 2.3 mm measurement beam of the autorefractor [17]. Moreover, in our study, we calculate that the 2.3 mm measurement beam would have remained entirely within the vision correction zone of the MOK-treated eyes out to an eccentricity of about 12°, but beyond that, the beam would have started to encroach on the molded treatment zone. These effects likely compromise the accuracy of the autorefractor measures made in this study.

In Figure A2, we have included corneal topography (tangential difference) maps (18-month topography minus baseline topography) for the MOK-treated eye with the greatest axial length increase over 18 months (+0.34 mm) and the map for the MOK-treated eye with the least axial length increase over 18 months (−0.48 mm). Figure A2 also shows maps for the fellow OK-treated eye in each case. Drawing general conclusions from these maps is problematic: they happen to show one case in which the lenses are well-centered (the eye with the least progression) and the other case in which the lenses are not well-centered (the eye with the greatest progression over 18 months). The visibility of the multifocal molding topography and the degree of lens centration varied markedly among participants (see link to Supplementary Materials below, which includes maps for all participants).

We recorded a rapid and sustained increase in choroidal thickness in MOK-treated eyes, whereas there was a sustained decrease in ChT in OK-treated eyes. Increased ChT has been associated with reduced eye growth [27,28] and so may have played a role in the reduced eye growth recorded in MOK-treated eyes over the course of the study. The reduced ChT that we observed in OK-treated eyes is contrary to the findings of most other studies, which report increases in choroidal thickness with conventional orthokeratology [22,29]. The reason for this inconsistency is not clear, although other studies have typically employed optical coherence tomography (OCT) to assess choroidal thickness, whereas we used ocular biometry (Lenstar) measures in the current study.

Between-eye comparison of VA between MOK and OK-treated eyes tended to show better VA in OK-treated eyes. This difference was significant at 6 months (MOK = 0.09, OK = 0.02 logMAR) and at 12 months (MOK: 0.09, OK 0.03 logMAR). The reduction in VA with MOK lenses may be related to the slight image degradation that can occur with multifocal optics [19]. For both lens types, Pelli–Robson contrast sensitivity decreased from baseline to 6 months (*p* < 0.05) and then increased again. There was no significant difference in CS between the two treatments at any point in the study.

The paired-eye design used in this study has a number of advantages in proof-of-concept investigations, and we have successfully employed it in a previous similar study of dual-focus soft contact lenses for myopia control [30]. A paired-eye design tends to naturally match baseline values of study parameters between control and experimental eyes because an individual’s two eyes are typically similar (e.g., similar refraction, axial eye length, etc.). When myopia progression occurs, the two eyes also typically progress at a similar rate. Therefore, the baseline progression rate is typically similar in both eyes of a paired-eye study, which is important when the progression rate is the primary outcome measure of the study. In contrast, recruiting participants in a parallel cohort study with matching baseline progression rate (as well as matching refraction, age etc.) in experimental and control arms is difficult. Moreover, a paired-eye design requires far fewer participants than a parallel cohort study with the same statistical power, which is partly because intra-subject variance in biometry measures is typically much less than inter-subject variance. A further advantage of a paired-eye design is that during the study, several potentially confounding factors that might influence progression are naturally matched between experimental and control eyes (e.g., parental myopia [31], time spent outdoors [32], hours of nearwork [33], etc.).

However, there are a number of limitations associated with this study, in addition to those associated with measures of refraction described above. Our measure of contrast sensitivity using the Pelli–Robson method may have underestimated the loss in contrast sensitivity with MOK treatment, as the method typically relies on lower spatial frequencies that may be less affected by the multifocal optics than higher spatial frequencies [34]. Another limitation relates to our use of ocular biometry rather than OCT to assess changes in choroidal thickness during the study. The ocular biometry waveform associated with the retina/choroid/sclera can be difficult to interpret and may be less accurate than OCT in this regard, and it is also limited to the subfovea. A further consideration is that we are unable to fully account for the shortening in AL, VCD, and IAL observed in the study. Some of the shortening in AL and VCD can be accounted for by increased ChT. However, we cannot rule out the possibility that the presence of multifocal optics may have compromised the accuracy of the ocular biometry measures. Although the paired-eye design has a number of advantages, the main limitation is that the participants did not wear the experimental lens in both eyes, as they would if the lenses were prescribed in practice. This difference may impact a number of practical outcomes. In the current case, one likely effect would relate to the reduced acuity associated with MOK lens wear that we recorded and whether this would influence wear compliance, patient satisfaction, etc.

## 5. Conclusions

This study showed that MOK lens wear is significantly more effective at slowing myopic eye growth in children than conventional OK over 18 months. In addition, MOK lenses appeared to slow the progression independently of the progression rate before the study period. Moreover, no additional negative side effects were found with MOK lenses apart from a slight reduction in VA equivalent to a loss of 2.3 Snellen letters.

## 6. Patents

M.L. and J.R.P. are named inventors on a patent for the MOK lens (Loertscher M and Phillips JR (inventors) Contact lens and method for prevention of myopia progression. Patent number US 9,753,309 (United States). Application number: 14/532,459. Status Published. Awarded date: 5 September 2017.)

## Figures and Tables

**Table 1:** Baseline measures (mean and standard deviation) of visual acuity, contrast sensitivity, photopic pupil diameter, refraction, myopia progression, and ocular biometry in eyes assigned to wear MOK lenses compared to those assigned to wear conventional orthokeratology (OK) lenses. Measures between eyes were compared with a paired t-test or Wilcoxon signed-rank test (WSRT).

Baseline Measure	MOK Eye Mean (SD) *n* = 30	OK Eye Mean (SD) *n* = 30	*p*
Visual Acuity (LogMAR)	0.01 (0.02)	0.00 (0.02)	0.59 (WSRT)
Pelli–Robson Contrast Sensitivity	1.63 (0.09)	1.64 (0.06)	0.50 (WSRT)
Pupil Diameter (mm)	4.74 (0.70)	4.81 (0.68)	0.436
Mean Sphere (D)	−2.72 (0.39)	−2.68 (0.80)	0.81
Progression in Last 6 Months (D/yr)	−0.96 (0.39)	−0.88 (0.34)	0.12 (WSRT)
Axial Length (mm)	24.57 (0.73)	24.53 (0.74)	0.237
Vitreous Chamber Depth (mm)	17.38 (0.79)	17.36 (0.79)	0.491
Anterior Chamber Depth (mm)	6.63 (0.22)	6.61 (0.21)	0.067
Inner Axial Length (mm)	24.28 (0.75)	24.24 (0.76)	0.262
Central Corneal Thickness (µm)	553 (34)	554 (32)	0.687
Choroidal Thickness (µm)	270 (61)	271 (63)	0.876

**Table 2:** Comparison of the effects of 18 months of MOK and OK lens wear on ocular structures. Changes over 18 months for eyes wearing MOK lenses and those wearing OK lenses. There was significantly less elongation of axial length, vitreous chamber depth, and inner axial length in MOK eyes than OK eyes. The central corneal thickness thinned significantly less in MOK eyes, but choroidal thickness increased significantly more in MOK eyes than in OK eyes.

Measure	Change Over 18 Months MOK Eye Mean (95% CI) *n* = 28	Change Over 18 Months OK Eye Mean (95% CI) *n* = 28	RGLM *p*
Axial Length (mm)	−0.044	0.129	0.001
	(−0.176 to 0.089)	(0.042 to 0.217)	
Vitreous Chamber Depth (mm)	−0.03	0.123	0.002
	(0.112 to 0.052)	(0.063 to 0.183)	
Anterior Chamber Depth (mm)	−0.006	0.019	0.065
	(−0.019 to 0.006)	(0.000 to 0.038)	
Inner Axial Length (mm)	0.005	0.151	0.022
	(−0.091 to 0.100)	(0.064 to 0.239)	
Central Corneal Thickness (µm)	−8	−13	0.005
	(−5 to −10)	(−9 to −16)	
Choroidal Thickness (µm)	31	−24	0.011
	(2 to 60)	(−57 to 8)	

## Data Availability

The data presented in this study are available on request from the corresponding author.

## Supplementary Materials

The original thesis, which describes the study in more detail, is publicly available at https://researchspace.auckland.ac.nz/handle/2292/22673. The thesis includes corneal topography maps before and after corneal molding and also tangential power differential maps (after fitting—pre fitting) for all MOK-treated eyes and all OK-treated eyes of 30 participants.
